# Permanent pacemaker post-valve surgery: Do valve type and position matter? A propensity score matching study

**Published:** 2021-11-29

**Authors:** Francesca Gatta, Yama Haqzad, Mahmoud Loubani

**Affiliations:** ^1^Cambridge University Hospitals, Cambridge, UK; ^2^Hull University Hospitals NHS Trust, Hull, UK

**Keywords:** pacemaker, valve surgery, mitral valve

## Abstract

**Background and Aim:**

This study evaluates whether aortic valve replacement (AVR) or mitral valve replacement (MVR) with biological versus mechanical prostheses is independent risk factors for permanent pacemaker (PPM) post-cardiac surgery, alongside traditionally accepted determinants.

**Methods:**

This study focused on single-centre retrospective analysis of 10 years of activity. Case–control 1-to-9 matching was performed for 7 pre-operative and 2 intraoperative confounding factors.

**Results:**

After matching, 617 patients were included for analysis: AVR (79.4% *n*=490) and MVR (20.6% *n*=127). PPM was implanted in 3.7% (*n*=18) and 3.1% (*n*=4), *P*=0.8, respectively. A further analysis for PPM rate in biological versus mechanical prostheses did not provide any significant result (*P*=0.6 AVR and *P*=0.8 MVR). Post-operative complications in AVR and MVR groups were as follows: Reopening (4.5% vs. 6.3%, *P*=0.4), myocardial infarction (0.8% vs. 3.2%, *P*=0.04), pulmonary (32.9% vs. 38.6%, *P*=0.3), neurological (9.2% vs. 11.8%, *P*=0.4), renal (9.8% vs. 7.9%, *P*=0.5), wound (1.4% vs. 2.4%, *P*=0.5), infective (5.5% vs. 8.7%, *P*=0.2), and multiple organ failure (4.9% vs. 5.5%, *P*=0.6). The length of intensive care unit (hours) and hospital stay (days) was 71±163.8 versus 106.5±243.7 (*P*=0.5) and 14.7±14.7 versus 18.9±20.8 (*P*=0.01). In-hospital mortality resulted in 4.1% for AVR and 3.9% for MVR, *P*=0.9.

**Conclusion:**

Valve position and valve type do not affect the likelihood of requiring permanent pacing in patients undergoing isolated aortic and MVR.

**Relevance for Patients:**

A significant proportion of patients undergoing cardiac surgery develop arrhythmias and conduction disturbances postoperatively, often requiring the implantation of a PPM. Determining factors associated with an increase likelihood of permanent pacing would allow the optimization of per- and intra-operative care, with the aim of reducing the incidence of patients requiring post-operative PPM insertion.

## 1. Introduction

Rhythm disturbances and conduction abnormalities significantly affect morbidity and mortality post-cardiac surgery and carry a financial impact due to the requirement of permanent pacing. The rate of permanent pacemaker (PPM) implantation ranges between 0.8 and 24% according to the type of cardiac surgery: Congenital heart surgery, heart transplantation, combined valve procedures with coronary artery bypass grafting (CABG) surgery, as well as tricuspid and mitral valve repairs, and a combination of either [1].

There are established additional risk determinants including pre-operative, intraoperative, and post-operative factors. Pre-operative predictors of PPM are older age, female sex, history of myocardial infarction, pre-existing rhythm and conduction disturbances, left ventricular systolic dysfunction, active endocarditis, New York Heart Association (NYHA) Class III–IV, and chronic kidney disease [1-3]. Intraoperatively, prolonged cardiopulmonary bypass and aortic cross-clamping times, as well as the use of intra-aortic balloon increase the likelihood of permanent pacing [4,5]. Postoperatively, electrolyte disorders and redo operations are associated with atrioventricular block, requiring PPM [6,7].

However, the literature provides little evidence about the risk of permanent pacing after isolated aortic or mitral valve replacement (MVR) with biological versus mechanical valves. This study sought to evaluate whether biological or mechanical prostheses in the aortic or mitral position individually increase the likelihood of permanent pacing.

## 2. Methods

### 2.1. Ethics

The study was registered as a departmental audit. Local institutional review board approval was granted by the audit department of Hull University Teaching Hospitals NHS Trust.

### 2.2. Database

This study is a single-center retrospective analysis of a series of 1357 consecutive patients undergoing isolated aortic valve replacement (AVR) and isolated MVR at the Cardiothoracic Department of Hull University Teaching Hospitals NHS Trust between January 2011 and December 2020.

All data were collected retrospectively and stored in our cardiac surgery database.

### 2.3. Study design and endpoint

The population was divided into two cohorts: Patients who underwent AVR and patients who underwent MVR.

Inclusion criteria were as follows: Any adult patient above the age of 18 years who underwent isolated aortic or MVR in Hull University Teaching Hospitals NHS Trust between January 2011 and December 2020.

Exclusion criteria were as follows: Concomitant CABG surgery, associated aortic surgery, multiple valve operation, and pre-operative PPM.

All patients in our cardiac unit follow an enhanced recovery protocol, including electrolyte monitoring and prompt correction of any disturbance, early extubation, short intensive care unit stay, and daily physiotherapy.

Specific subgroups were created for the analysis of PPM implantation in relation to biological versus mechanical prostheses. AVR and MVR cohorts were further divided into two groups: Tissue AVR and mechanical AVR, tissue MVR and mechanical MVR.

The primary outcome was the incidence of post-operative PPM.

Secondary outcomes included reoperation, perioperative complications (myocardial infarction, pulmonary, renal, neurological, wound, and infective), multiple organ failure, intensive care units (intensive therapy unit) stay, hospital stay, and in-hospital mortality.

### 2.4. Propensity matching and statistical analysis

To minimize the bias related to the well-established risk factors for permanent pacing, and analyze exclusively the individual determinants objectives of this study, a propensity score match was run. Due to the uneven number of patients, a case–control matching 1:9 (MVR: AVR) with a 0.01 tolerance was performed for the following variables:

- Pre-operative factors: Age, sex, body mass index, NYHA class, history of ischemic heart disease, chronic kidney disease, and diabetes mellitus
- Intraoperative factors: National Confidential Enquiry into Patient Outcome and Death class and intra-aortic balloon pump.

Categorical variables were reported as frequency and percentage, while continuous variables were reported as mean ± SD.

Statistical significance for the analysis was defined at *P*<0.05.

For non-parametric variables, Pearson Chi-square tests were performed; for parametric variables, the one-way ANOVA test was run.

The statistical analysis was performed on SPSS System for statistics by two authors independently.

## 3. Results

### 3.1. Patient characteristics

**Figure 1:**
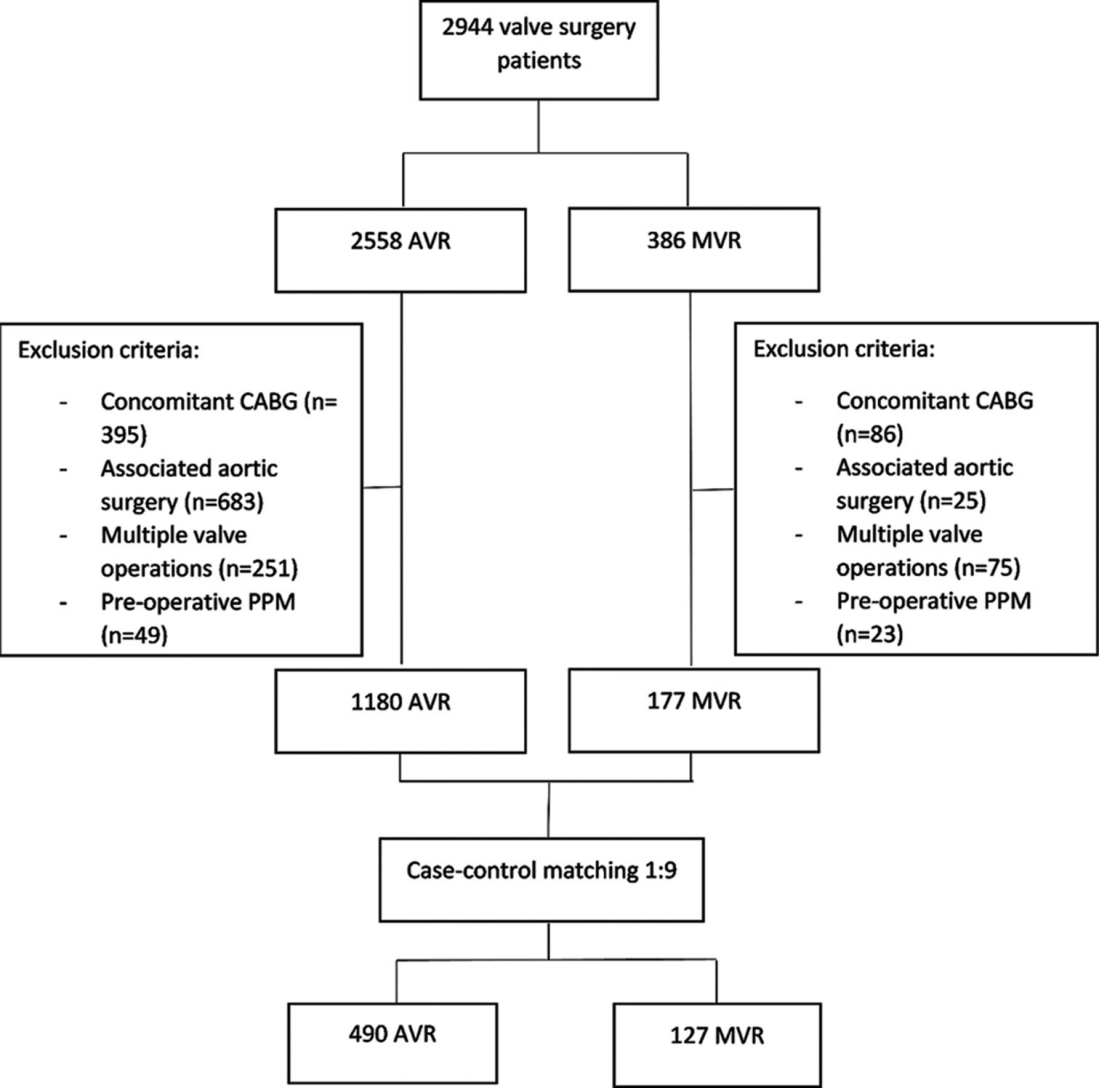
Patient selection and case–control matching

To the original database of 1357 patients, 86.9% (*n*=1180) AVR and 13.1% (*n*=177) MVR, case–control matching was applied, resulting in 617 patients, 79.4% (*n*=490) AVR and 20.6% (*n*=127) MVR (Figure 1).

Population demographics for AVR and MVR are shown in Table 1. Mean age was 66.8±12.4 and 66±11.3, *P*=0.5. Male patients were 50.8% (249) and 52% (315), *P*=0.8.

**Table 1:** Patient characteristic

	AVR (*n*)	MVR (*n*)	*P*-value
*n*	79.4% (490)	20.6% (127)	
Male	50.8% (249)	52% (315)	0.8
Age	66.8±12.4	66±11.3	0.5
BMI	27.4±4.8	26.8±5.6	0.2
Hypertension	62.7% (307)	55.9% (71)	0.2
Diabetes mellitus	11.6% (57)	10.2% (13)	0.7
COPD	19.4% (95)	18.9% (24)	0.9
Chronic kidney disease	3.1% (15)	2.4% (3)	0.7
Neurological history	13.1% (64)	12.6% (16)	0.9
Ischemic heart disease	11.2% (55)	9.4% (12)	0.6
Non-sinus rhythm	26.9% (132)	40.9% (52)	0.01
- Atrial fibrillation	25.9% (127)	32.4% (41)	
- AVB I	0.8% (4)	8.5% (11)	
- AVB II (Mobitz 1)	0.2% (1)	0	
Left ventricle EF			0.01
- >49%	68.4% (335)	59.9% (76)	
- 30–49%	24.5% (120)	37% (47)	
- <30%	7.1% (35)	3.1% (4)	
Active endocarditis	5.9% (29)	11.8% (15)	0.02
N previous heart surgery	0.09±0.3	0.2±0.4	0.02
Valve pathology			<0.001
- Stenosis	66.1% (324)	3.1% (4)	
- Regurgitation	16.1% (79)	81.1% (103)	
- Mixed	17.8% (87)	15.7% (20)	
NYHA			0.5
- Class I/II	50.2% (246)	46.5% (59)	
- Class III/IV	49.8% (244)	53.5% (68)	
EuroSCORE II	5±5.4	4.9±6.1	0.8
Critical status	2.7% (13)	6.3% (8)	0.04
NCEPOD			0.9
- Elective	74.1% (363)	71.7% (91)	
- Expedite	3.7% (18)	3.2% (4)	
- Urgent	21.4% (105)	24.4% (31)	
- Emergency	0.8% (4)	0.8% (1)	
IABP	4.9% (24)	7.1% (9)	0.3
Operation time (min)	251.1±57.4	268.7±63.5	0.01

Chronic kidney disease defined as GFR<60 ml/min/1.73 m^2^. Neurological history included previous stroke or known carotid artery stenosis. BMI: Body mass index; COPD: Chronic obstructive pulmonary disease; AVB I: First-degree atrioventricular block; AVB II: Second-degree atrioventricular block; EF: Ejection fraction; NCEPOD: National Confidential Enquiry into Patient Outcome and Death; IABP: Intra-aortic balloon pump.

The presence of comorbidities varied slightly in the two cohorts. Mitral patients reported a higher degree of pre-operative non-sinus rhythm (40.9% vs. 26.9%, *P*=0.01) and active endocarditis (11.8% vs. 5.9%, *P*=0.02). Conversely, a greater proportion of aortic patients had either a good (>49%) or poor (<30%) left ventricle systolic function, whilst the MVR group had a higher percentage of moderate (30–49%) systolic dysfunction (*P*=0.01).

The valve pathology differed significantly. Whilst majority of patients undergoing AVR presented with stenosis, those requiring MVR were diagnosed with regurgitation. Stenosis accounted for 66.1% in AVR and 3.1% in MVR, regurgitation was 16.1% and 81.1%, mixed stenosis-regurgitation 17.8% and 15.7%, *P*>0.001, respectively.

Intraoperatively, mitral valve patients had a prolonged cumulative operation time, 251.1±57.4 min versus 268.7±63.5 min for AVR, *P*=0.01. Finally, valve type and size are listed on Table 2. As expected, these differ in both groups (*P*<0.001).

**Table 2:** Valve type and size in AVR and MVR

	AVR (*n*=490)	MVR (*n*=127)	*P*-value
Valve type, % (*n*)			<0.001
- ATS	3.7 (18)	5.7 (8)	
- Carboseal	7.6 (37)	11.3 (14)	
- Edwards	17.7 (87)	1.7 (2)	
- Hancock	26.9 (132)	37.3 (47)	
- Mitroflow	1.4 (7)	0	
- On-X	15.8 (77)	15.8 (20)	
- Perceval	1.6 (8)	0	
- St. Jude Medical	3.7 (18)	3.4 (4)	
- St. Jude	0	24.8 (32)	
- Trifecta	21.6 (106)	0	
Valve size, % (*n*)			<0.001
- 18–20	6.8 (33)	0	
- 21–23	59.8 (293)	0	
- 25	24 (118)	10.4 (13)	
- 26–27	7 (34)	19.4 (25)	
- 28–30	2.4 (12)	25.5 (31)	
- 31–33	0	44.7 (58)	

### 3.2. Primary outcome

There was no statistically significant difference in PPM implantation in AVR versus MVR (Table 3). About 3.7% (*n*=18) of patients undergoing AVR required permanent pacing versus 3.1% (*n*=4) of patients in the MVR cohort, *P*=0.8. The post-operative arrhythmias, date of implantation, and indication for PPM are also reported on Table 3. The latter was complete heart block in all patients.

**Table 3:** Permanent pacemaker in the AVR and MVR groups

	AVR (*n*=490)	MVR (*n*=127)	*P*-value
Post-operative arrhythmias, % (*n*)			<0.001
- Atrial fibrillation	51.4 (252)	61.6 (78)	
- High degree AV block	3.7 (18)	3.1 (4)	
- Ventricular tachycardia	0.7 (3)	3.4 (4)	
PPM indication, % (*n*)			0.8
- CHB	3.7 (18)	3.1 (4)	
PPM, % (*n*)			0.8
- Yes	3.7 (18)	3.1 (4)	
- No	96.3 (472)	96.9 (123)	
PPM implantation, % (*n*)			0.4
- Before day 5	0.4 (2)	0	
- After day 5	3.3 (16)	3.1 (4)	

AV: Atrioventricular; CHB: Complete heart block.

There was also no statistically significant difference in PPM implantation in mechanical versus biological valves in either AVR or MVR patients. Patients with an aortic valve pathology required a mechanical valve in 33.7% (*n*=165) of cases and a biological valve in 66.4% (*n*=325). Conversely, the majority of patients undergoing MVR had a mechanical prosthesis implanted (57.5% *n*=73) as opposed to a biological valve (42.5% *n*=54). The incidence of PPM in the AVR group resulted in 4.2% (*n*=7) for mechanical valves and 3.4% (*n*=11) for biological valves, *P*=0.6 (Table 3). The rate of permanent pacing in the MVR group was 2.7% (*n*=2) for mechanical prostheses and 3.7% (*n*=2) for biological ones, *P*=0.8 (Table 4).

**Table 4:** Permanent pacemaker implantation: tAVR versus mAVR and tMVR versus mMVR

	PPM	*P*-value
AVR, % (n)		0.6
- tAVR	3.4 (11)	
- mAVR	4.2 (7)	
MVR, % (n)		0.8
- tMVR	3.7 (2)	
- mMVR	2.7 (2)	

tAVR: Tissue aortic valve replacement, mAVR: Mechanical aortic valve replacement, tMVR: Tissue mitral valve replacement, mMVR: Mechanical mitral valve replacement

Patients with a newly implanted PPM did not report any procedure-related early complications.

### 3.3. Secondary outcome

The main endpoint of the study was the incidence of PPM insertion. However, other post-operative complications and short-term mortality of patients undergoing aortic or MVR were also analyzed.

Clinical outcomes are listed on Table 5. Whilst both groups had similar rates of post-operative complications and short-term mortality, mitral patients reported a higher incidence of myocardial infarction (3.2% for MVR vs. 0.8% for AVR, *P*=0.01) and required a prolonged hospital stay (18.9±20.8 days for MVR vs. 14.7±14.7 days for AVR, *P*=0.01). Such results may be due to the higher proportion of mitral patients with active endocarditis.

**Table 5:** Secondary outcomes

	AVR (*n*)	MVR (*n*)	*P*-value
Reopening	4.5% (22)	6.3% (8)	0.4
Myocardial infarction	0.8% (4)	3.2% (4)	0.04
Respiratory complications	32.9% (161)	38.6% (48)	0.3
Neurological complications	9.2% (45)	11.8% (15)	0.4
Renal complications	9.8% (48)	7.9% (10)	0.5
Wound complications	1.4% (7)	2.4% (3)	0.5
Infective complications	5.5% (27)	9.7% (11)	0.2
MOF	7.9% (24)	5.5% (5)	0.6
ITU stay (min)	71±163.8	106.5±243.7	0.5
Hospital stay (days)	14.7±14.7	18.9±20.8	0.01
In-hospital mortality	4.1% (20)	3.9% (5)	0.9

MOF: Multiple organ failure; ITU: Intensive therapy unit.

## 4. Discussion

A significant proportion of patients undergoing cardiac surgery develop arrhythmias and conduction disturbances postoperatively, often requiring the implantation of a PPM. About 2–4% of patients requiring isolated valve surgery need a PPM due to complete or high-degree AV block [4]. Determining factors associated with an increase likelihood of PPM insertion would allow the optimization of per- and intra-operative care, with the aim of reducing the incidence of patients requiring post-operative PPM insertion.

Alongside the traditional risk factors for permanent pacing in valve surgery, the close proximity of operative procedures to any part of the conduction system may cause its direct or indirect injury. This results in different degree of atrioventricular block, ultimately leading to bradyarrhythmias and PPM [2]. Specifically, the surgical manipulation associated with aortic valve surgery predisposes to a higher incidence of such damage, as the AV bundle runs on the septum, next to the aortic annulus. Several studies report a second-degree block in 12.4–44% of patients undergoing valve surgery and a third-degree heart block in 42.3–87.5% [7,8]. In our analysis, the indication for PPM was complete heart block for all patients.

A 5-fold increase in the requirement for PPM is observed in surgery with severely calcified aortic stenosis and/or involving the tricuspid valve [4]. Dawkins *et al*. demonstrated the significant contribution of aortic valve diseases, including stenosis and regurgitation, in the requirement of permanent pacing [9]. Similar results are shown in other recent studies [3,5]. In addition, Erdogan reported aortic annular calcification was proven to increase the likelihood of PPM (*P*<0.001, odds ratio [OR] 0.05, 95% confidence interval [CI] 0.01–0.24) [10]. This is particularly relevant in aortic root replacement when the aortic valve morphology is bicuspid (*P*=0.02, OR 0.24, 95% CI 0.07–0.84) [11]. Merin *et al*. demonstrated an increased risk of permanent pacing in patients undergoing AVR, in comparison with mitral and tricuspid interventions (*P*<0.0001) [3].

For mitral valve surgery, several studies reported a variable rate of PPM, ranging from 2.6% to 7.7%, with no significant relation to the valve approach (trans-septal, superior trans-septal, and conventional left atriotomy) [12,13]. No research has been conducted on the impact the type of prosthesis might have on the rate of PPM.

Recently, Moskowitz conducted a comprehensive analysis of permanent pacing requirement following valve surgery in a large population size. Combined AVR and MVR carried the highest risk (13.3%), compared with isolated AVR and MVR, as well as MV repair (*P*>0.001). Of these, the MVR group followed with the second highest risk (10.5%) [14]. Although our report does not analyze combined valve surgery, patients requiring isolated MVR had a similar incidence of PPM to isolated AVR. Nevertheless, in our population, the incidence of permanent pacing was low.

In our study, patients were propensity score matched for the well-known permanent pacing risk factors, and isolated aortic and MVRs compared. Specific pre-operative and operative factors were not matched to portray a realistic dataset; these included active endocarditis, valve pathology, left ventricular function, and pre-operative rhythm. Furthermore, biological and mechanical prostheses were analyzed in both groups to detect a possible influence on PPM implantation. Neither valve position nor valve type appeared to be additional isolated determinants for permanent pacing.

## 5. Conclusion

Whilst several pre-operative and intraoperative factors have been traditionally associated with an increased rate of PPM implantation, valve position and valve type do not affect the likelihood of requiring permanent pacing in patients undergoing isolated aortic and MVR.

### Limitations

This study presents some limitations.

It is a retrospective single-center analysis and therefore results may not be generalizable. Moreover, patients were recruited over a long time period of 10 years, over which surgical practice might have changed, potentially influencing outcomes. Nevertheless, the large sample size and the meticulous statistical analysis should reduce the potential for bias.

The population was matched with a 9:1 case–control method. This may carry more bias than the traditional 1:1 propensity score matching, as the additional matches could have a lower quality than the first match identified.

Finally, not all pre-operative and intraoperative factors were propensity matched. Only those traditionally accepted as determinants for permanent pacing were matched, with the aim to convey a database still reflective of clinical practice.

### Conflicts of Interest

The authors declare no conflicts of interest.
